# A Smart Context-Aware Hazard Attention System to Help People with Peripheral Vision Loss

**DOI:** 10.3390/s19071630

**Published:** 2019-04-05

**Authors:** Ola Younis, Waleed Al-Nuaimy, Fiona Rowe, Mohammad H. Alomari

**Affiliations:** 1 Department of Electrical Engineering and Electronics, University of Liverpool, Liverpool L69 3GJ, UK; wax@liverpool.ac.uk; 2 Department of Health Services Research, University of Liverpool, Liverpool L69 3GL, UK; rowef@liverpool.ac.uk; 3 Department of Computer Science, University of Liverpool, Liverpool L69 3BX, UK; alomari@liverpool.ac.uk

**Keywords:** hazard detection, context-aware, wearable assistive technology, object detection, object tracking, hazard perception, augmented reality, Kalman filter

## Abstract

Peripheral vision loss results in the inability to detect objects in the peripheral visual field which affects the ability to evaluate and avoid potential hazards. A different number of assistive navigation systems have been developed to help people with vision impairments using wearable and portable devices. Most of these systems are designed to search for obstacles and provide safe navigation paths for visually impaired people without any prioritisation of the degree of danger for each hazard. This paper presents a new context-aware hybrid (indoor/outdoor) hazard classification assistive technology to help people with peripheral vision loss in their navigation using computer-enabled smart glasses equipped with a wide-angle camera. Our proposed system augments users’ existing healthy vision with suitable, meaningful and smart notifications to attract the user’s attention to possible obstructions or hazards in their peripheral field of view. A deep learning object detector is implemented to recognise static and moving objects in real time. After detecting the objects, a Kalman Filter multi-object tracker is used to track these objects over time to determine the motion model. For each tracked object, its motion model represents its way of moving around the user. Motion features are extracted while the object is still in the user’s field of vision. These features are then used to quantify the danger using five predefined hazard classes using a neural network-based classifier. The classification performance is tested on both publicly available and private datasets and the system shows promising results with up to 90% True Positive Rate (TPR) associated with as low as 7% False Positive Rate (FPR), 13% False Negative Rate (FNR) and an average testing Mean Square Error (MSE) of 8.8%. The provided hazard type is then translated into a smart notification to increase the user’s cognitive perception using the healthy vision within the visual field. A participant study was conducted with a group of patients with different visual field defects to explore their feedback about the proposed system and the notification generation stage. The real-world outdoor evaluation of human subjects is planned to be performed in our near future work.

## 1. Introduction

Blindness and impaired vision result from a range of causes including glaucoma, cataract, and age-related macular degeneration [1]. While the latter causes central vision loss, the former affects mainly the outer visual field and Peripheral Vision (PV) in particular [2]. While most visual acuity problems are correctable using different techniques and traditional solutions such as eyeglasses, visual field defects are not easily rehabilitated. This is because most of these defects happen after brain injury or eye conditions where parts of the visual system become permanently diseased [3].

**Figure 1:**
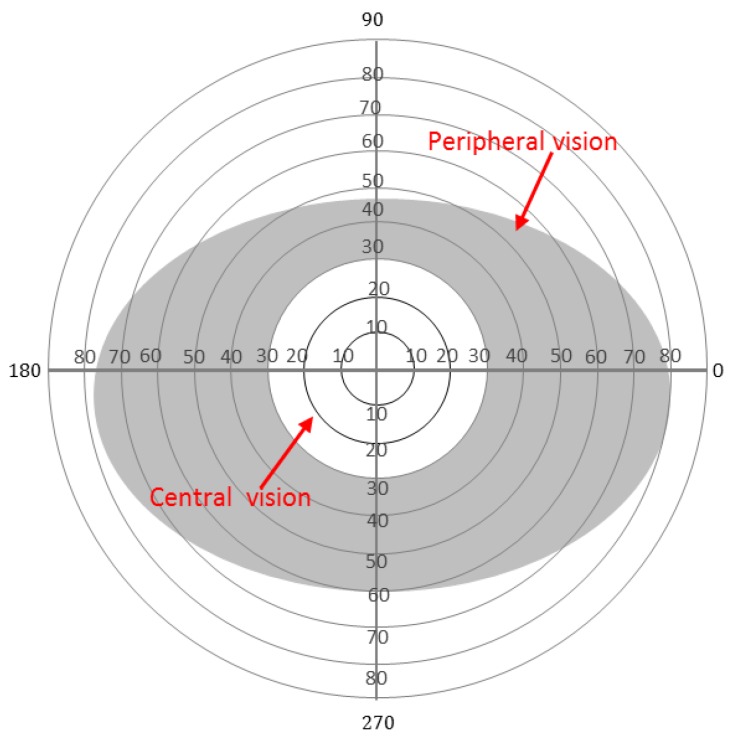
The healthy visual field extent for both eyes. The white circle in the middle represents the central visual field, while the grey area represents the peripheral visual field.

Two types of vision areas define a human’s visual field: central and peripheral. These areas are used to see and recognise different levels of details and information. Our brain uses the central visual (5°) field most for reading, focusing, drawing, crossing the road, and many other daily activities that require a deep understanding of specific details. On the other hand, the peripheral vision is used to detect larger contrasts, colours and motion and extends up to 160° horizontally and 145° vertically for each eye [4]. While the peripheral vision is inferior to the central vision in terms of detailed view, it is particularly useful to attract the brain’s attention for the surrounding environment. One of the critical roles that a human’s peripheral vision provides is the ability to detect and avoid potential hazards in the surroundings. To explore the fine details about a specific object, humans use head movements to gather more information and increase their cognitive understanding. Standard visual field extensions for both eyes are shown in Figure 1. The central vision is shown as a white circle in the middle covering 30° around the fixation point (assumed to be the centre of the figure). Due to retinal eccentricity [5], different degrees of resolution occur in different parts of the visual field areas. The more central the area, the more resolution for vision [6].

**Figure 2:**
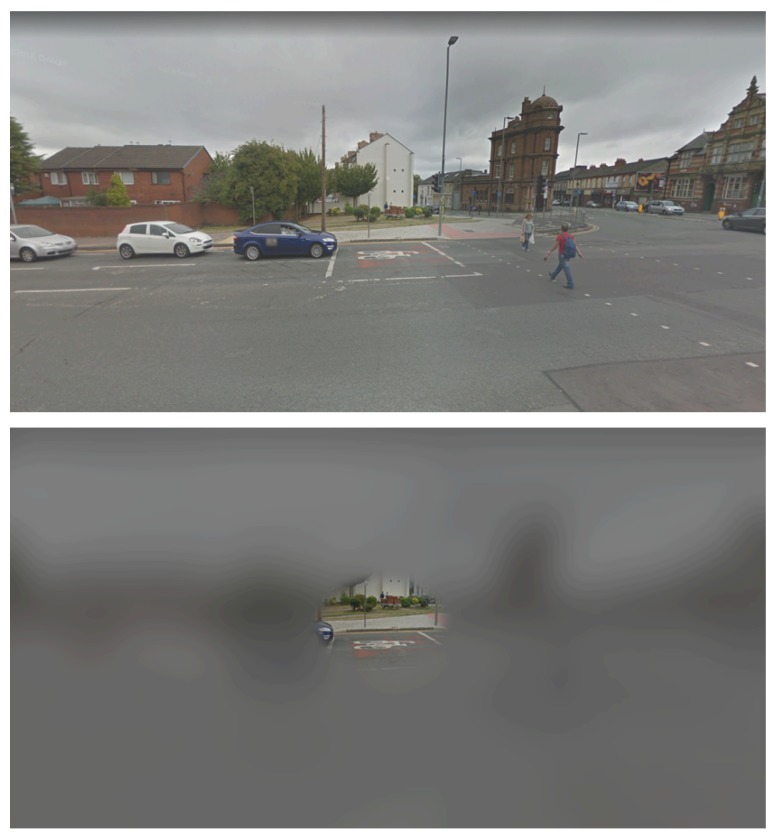
A simulated view for the healthy vs. tunnel vision might appear. The top picture shows the healthy vision; the bottom image shows how a person with tunnel vision might see the same scene.

In the case of peripheral vision loss, the outer visual field areas are impaired to varying degrees, while central vision may remain healthy. Tunnel vision is one form of peripheral vision loss and the most extreme case. People with tunnel vision can see through a tiny circular area in their central vision (≈10°). In this case, it is essential for the person to continuously shift their focus around to have a full understanding of the surroundings and possible threats [7,8]. Figure 2 shows a simulated view of the same scene with healthy and tunnel vision.

People with peripheral vision loss have both healthy and defected vision areas in their visual field. Eye specialists use a perimetry test to measure these areas. The test clearly defines where the person can and can’t see. It can also plot the progression of field loss over time [9].

Designing a system that implements computer vision algorithms in real time to provide useful information about any possible threats existing in the user’s blind area will enhance functional vision by giving cues from the affected field without the need for shift of their fixation point all the time. It is essential that the additional cues provide fast and trustful notification that reflects the hazard type, danger degree and, most importantly, the location of that hazard.

Smart Assistive Technologies (AT) and mobile healthcare systems are developing rapidly. With the massive growth in the hardware and software sectors, wearable smart devices have become widely affordable. Vision assistance devices have been developed to be worn on several body parts such as the head, chest, fingers, feet, and ears. Digital cameras have been used to collect data about the surrounding environment and process this to generate useful outputs for many vision rehabilitation applications such as indoor/outdoor navigation, obstacle detection and tracking and activity recognition. All these applications have been developed with the common goal to enhance the individual’s quality of life [10].

Object recognition, object tracking, visual odometry, activity classification and many other real-time computer vision-related algorithms are now used every day in several applications like video surveillance, AT, video compression and robotic navigation. These applications are becoming more affordable in the healthcare field due to the considerable developments for mobiles and portable smart devices [11].

Traditional computer vision applications use the captured data to respond in a real-time manner to a specific condition or scenario. On the other hand, context-aware systems try to understand the context and circumstances of a given case and respond or update their response accordingly [12]. This is applied not only in the system design phase but also in the on-going processing time while the system is performing its tasks [13].

Context-Aware Assistive Systems (CAAS) have become widely used in autonomous cars, mobile phone applications and healthcare sectors [14]. Using the context awareness concepts with computer vision algorithms and new wearable technology could provide smart, context-aware and easy to use wearable AT for the visually impaired people. Incorporating such context-awareness assistive software into wearable technology suitable for use by the visually impaired requires careful consideration and a delicate balance between computation power, visual display and usability/wearability.

While Virtual Reality (VR) attempts to replace the user’s vision with a computer-generated, virtual environment [15], Augmented Reality (AR) and Mixed Reality (MR) [16] use the individuals’ vision to add more helpful information and extend their knowledge without blocking the original vision [15,17]. In AR applications, the computer-generated inputs are not able to interact with the real world content, but, in MR applications, they can react to each other.

In this paper, we propose a smart assistive technology using smart glasses and computer vision algorithms; a system that recognises objects in the user’s visual field and classifies them to determine the possible danger level is designed. Motion features for the detected objects such as the speed, direction, location and age (the appearance time in terms of the number of frames) were extracted using object tracking modules. This work is part of a larger project to developing a user-centred design for a wearable, context-aware hazard detection system for people with peripheral vision loss [18,19].

A Neural Network classifier is implemented to classify the detected objects based on the extracted motion features into one of five classes. Public and private datasets are used to train the system, with a predefined ground truth labelled by an expert. This is used to generate a meaningful notification that is reliable and in the best visual position to warn the person about any possible hazard. The contributions of this paper can be summarised as follows:

1. A context-aware assistive technology is developed to increase cognitive awareness for people who have vision impairment using computer vision and machine learning algorithms.
2. An egocentric indoor and outdoor hazard recognition dataset is created using a wearable camera and classified using deep learning object detector and Kalman Filter tracker to be used in the hazard detection and classification for people with vision defects.
3. A motion model that describes the hazard type in the user’s environment based on motion features is presented to be used in the classification stage.
4. A machine learning-based hazard classification system using motion features for multiple hazards simultaneously is proposed to provide a smart and early warning system to help people with peripheral vision loss.

This paper is structured as follows: in Section 2, a review of the related literature is presented, followed by a description of our proposed system. Section 4 describes the used datasets employed while system evaluation experiments are presented and analysed in Section 5. Finally, research findings, conclusions, and recommendations for future work are provided in Section 6.

## 2. Literature Review

### 2.1. Vision-Based Systems

As computer vision algorithms, sensor technologies and hardware have been used together, the idea of developing wearable or portable assistive technologies for visually impaired people evolved. These technologies were applied to deliver both obstacle detection/avoidance and indoor navigation systems. The primitive version of these systems used basic image processing and computer vision techniques, while the recent versions are smart enough to draw a safe path for user navigation [20].

In 2001, a group at Harvard developed a device that produced AR vision for people with severe peripheral vision loss (tunnel vision) [21]. The device comprises a wide-angle camera and one display unit that projects a processed image (cartoon style) from the camera on the regular (healthy) vision area. The device was tested on healthy and vision impaired people and the results showed improvements of self-navigation and object finding. However, this solution created a double vision that could cause distraction and reduce the efficiency of a healthy vision.

In 2007, the Stereo Vision-based Electronic Travel Aid (SVETA) system was proposed to help blind people in their navigation [22]. The authors used a stereo camera to capture the surrounding environment and determine the location and distance of the obstacles in the user’s navigation path. The output of the proposed system is delivered through stereo earphones. The input sensor and the output unite are all connected to a compact computing device that is placed in a specially designed pouch. While the system’s tests proved its applicability in helping blind users, outdoor environment challenges and the slow performance could be considered as critical issues for the presented method.

Elango and Murugesan [23] proposed their work of using AR to extend visually affected patients’ knowledge using the Cellular Neural Network (CNN). The study presented a model consisting of a monochrome camera, display unit and a portable processor to perform image processing. The main limitation of this work is the simplicity of the generated information (edge detection with a scene reduction) that are displayed on the top of the user’s real-world images.

In 2014, Fiannaca et al. presented Headlock [24], a wearable device to assist blind people in traversing open spaces. The system used Google glasses and OpenCV blob detection algorithm to detect doors and guide the blind person towards it with minimum veering and the shortest path. Although the presented work provided quantitative and qualitative results after testing the system’s usability with blind subjects, limiting the object detection and navigation to doors only make it inefficient for hazard avoidance or general blind navigation systems.

In the same year, a group of researchers from Switzerland proposed a new computer vision-based system to help rollator users in their indoor and outdoor navigation. Using 3D and stereo data, they implemented two obstacle detectors to capture any possible danger in the user’s pathway [25]. They also introduced a new way to enhance the distorted 3D objects by using the pose estimation technique for the combined 3D points. Although this work could help visually impaired people in their navigation, the detection stage (the core module in work) does not work in sophisticated scenarios such as multiple moving objects. In addition to this, no motion compensation methods were presented to overcome the camera movement.

More recently, a group of researchers from Munich developed a lightweight device to help visually impaired people during their everyday activities [26]. This wearable device uses two depth cameras for data collection and a real-time depth processing algorithm extracts information from the video stream to produce acoustic outputs. The use of this low power, low latency sensor is useful to develop a user-friendly device that performs real-time processing. However, the clinical tests for this system revealed that real-life scenarios are far more complicated and need more sophisticated systems and algorithms to deal with dynamic motion and multiple object detection.

Obstacle Stereo Feedback (OSF) system was implemented using a depth sensor and computer vision algorithms to guide blind or impaired vision users in indoor navigation [27]. The hand-free system uses depth information for obstacles in front of the user and produces acoustic notification when necessary. The developers also provided Head-Related Transfer Functions (HRTF) to their system to create a more realistic and 3D stereo sound environment that represents the detected obstacles. Moving objects were not tracked in this system. This limits the detection stage to stationary obstacles only. In addition, the user’s motion was not considered in this work. This could affect the accuracy of hazard detection in general.

In 2017, a comprehensive review was presented by Elmannai and Elleithy [28]. In their study, the authors demonstrated the progress of wearable and portable devices for impaired vision people and how the technologies have been developed over the years to help in this problem. The survey discussed the technology limitations, challenges, directions and future possibilities for this field. The authors concluded with a list of essential guidelines needed to develop assistive technology for blind people such as performance, wireless connectivity, reliability, simplicity, wearability and economic accessibility.

Detecting traversable area and avoiding obstacles for visually impaired people was proposed by Yang et al. [29]. The authors presented a sensor combination, multi-thread assistance framework integrating wearable smart glasses, Inertial Measurement Unit (IMU) sensor, and the Intel RealSense RS410 depth camera. Although the proposed work enhanced the pathfinding task for blind and visually impaired people, the system did not provide any information about the type of the detected objects or the motion model of the dynamic objects in the user’s environment.

Most of the systems mentioned above focused on technical aspects and assumed the users to be blind. Partially seeing users use their healthy vision as much as they can rather than use any device to facilitate social acceptability. The need for a wearable AT that is unobtrusive with physical convenience and utilises the healthy vision for the peripheral vision loss is highly needed.

### 2.2. Context-Aware Systems

In 1999, Abowd et al. [30] introduced the concept of context-awareness in computing. They described it as the ability of computer systems to simulate real human communication options by gathering data about its surrounding environment at any given time. In addition, these systems should adjust their interactions based on the collected data accordingly. Context-aware approaches use software and hardware for data collection. They perform real-time data analyses and processing smartly.

Recently, a context-aware indoor mapping system was developed by a group of researchers [31] based on Tango devices, semantic maps editors, and obstacle detection algorithms. This system (ISANA; Intelligent Situation Awareness and Navigation Aid) uses information from the mentioned modules to compute a safe navigation path for blind people.

A novel navigation assistant system for blind people was implemented in the work proposed by Tapu et al. [32]. The proposed system (denoted DEEP-SEE) detects both moving and stationary objects using the You-Only-Look-Once (YOLO) object recognition method [33]. Based on two convolutional networks, their system tracks the detected objects in real time and solves the occlusion problem. The system then classifies the object based on its location, type and distance and notifies the user using acoustic warning message prioritisation based on the object semantic interpretation.

ARIANNA [34] is a path recognition system used to help in the indoor navigation for people with impaired vision. It is a flexible application that could be used on smartphones and portable devices with AR capabilities. This system uses computer vision algorithms to detect tapes deployed in the users’ pathway to easily navigate them through the use of vibration signals as feedback. The authors also presented a second version for their system by enhancing the tracking performance [35].

A novel and smart indoor mobile AT for blind people using Tango devices was introduced by Li et al. [36]. They delivered a full system from obstacle detection and tracking phases to the final notification. The system uses the indoor map editor to extract semantic features from the geometric map for global path mapping. This step continuously updated and enhanced in real time with the obstacle detection and avoidance algorithm they created to correct the projected obstacles path if the user faces any possible threat. Finally, a smart cane prototype was designed and implemented for human–machine interface and communication.

From the previously mentioned studies, we can clearly see the shortage of outdoor context-aware hazard detection systems for impaired vision people. Moreover, most of these proposed systems assume the total blindness of the user’s vision. People with peripheral vision loss can retain healthy vision with good central acuity. This highlights the need for smart outdoor hazard detection and classification systems that work in real time and deliver smart notifications that could alert the user as early as possible about developing hazards.

## 3. The Proposed System

We propose a wearable AT for Visually Impaired People (VIP) providing early smart notifications for potential hazards. The proposed technology is to be used with wearable smart glasses that utilise a wide-angle camera integrated into an Android device.

**Figure 3:**
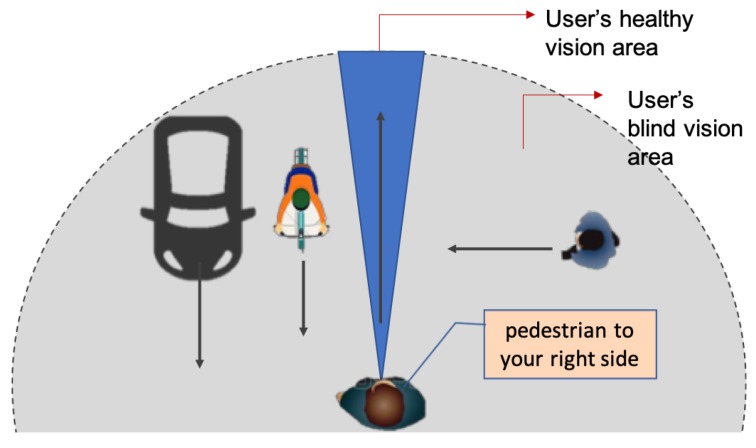
The conceptual representation of our system.

Rather than replace their (already limited) visual field, this system is to help VIP compensate for their visual defects by increasing their cognitive awareness about the peripheral field. The purpose is to alert the user to any possible hazards or threats in their environment. Figure 3 shows the general concept in this work. In this figure, the grey area represents the user’s peripheral vision (blind), while the blue area represents the user’s actual vision. People with peripheral vision loss miss the information that describes their surrounding. Therefore, they could not build a mental map of the physical environment. Although it is possible to use other sensory data as sound and smell, the vision sensor is valuable to determine dangerous situations during navigation. The system will provide visual feedback for the user by detecting, tracking and classifying the objects in the blind area and generating a suitable notification to increase user’s awareness.

### 3.1. User Requirements

An exploratory study was conducted with five visually impaired participants to understand the daily challenges and needs for VIP. The participants answered a questionnaire including questions about the significant challenges they face that would affect their quality of life in general and their independent navigation in particular.

**Figure 4:**
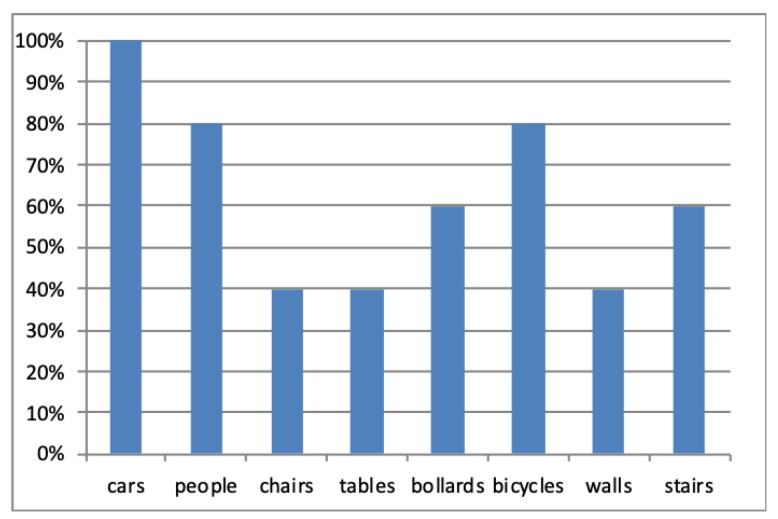
Users’ preferences for the objects’ types they would like to have information.

It was found that 80% of the participants prefer having notifications about moving rather than stationary objects. When they were asked to specify the type of objects they are interested in, cars, people and bicycles were the most chosen options. Figure 4 shows the users’ preferences for the types of objects they would require a notification.

Several research papers explored the VIP requirements for assistive technologies. In their research paper [37], Jafri and Khan presented their obstacle detection and avoidance application for VIP based on the results they got from a semi-structured interview. While the human guide was superior to the white cane as a navigation aid, the participants mentioned that this method causes them problems as they depend entirely on the guide who may not provide an accurate warning about obstacles. In the same study, moving and minimal obstacles were the most difficult to detect and avoid during the indoor navigation.

The lack of information that describes the physical environment is one of the core challenges for VIP navigation. This was mentioned by many participants as the need for a clear description for indoor and outdoor main landmarks that would help them build a mental map [38,39]. In their comprehensive study about computer vision algorithms for AT, Leo et al. [40] mentioned several open challenges for developing AT for VIP. Object detection and tracking problems are examples, especially for egocentric video streams.

Based on these requirements, preferences and challenges, we developed our design in a smart way that would provide early, meaningful and straightforward notifications for extending the user’s mental map. We define the possible hazard by any moving or stationary object in the users’ pathway that they are not able to see or recognise and may collide with while walking. Therefore, the system starts scanning the real-time video to search for objects and then tracks their movement. Motion features such as object speed, direction and location in addition to the object type and other features decide the level of danger for each detect hazard. As shown in Figure 3, the goal of the system is to produce notifications for the user just to become aware of potential hazards. In this case, the system prioritises the detected hazards to generate useful feedback without overloading the user with too much information.

**Figure 5:**
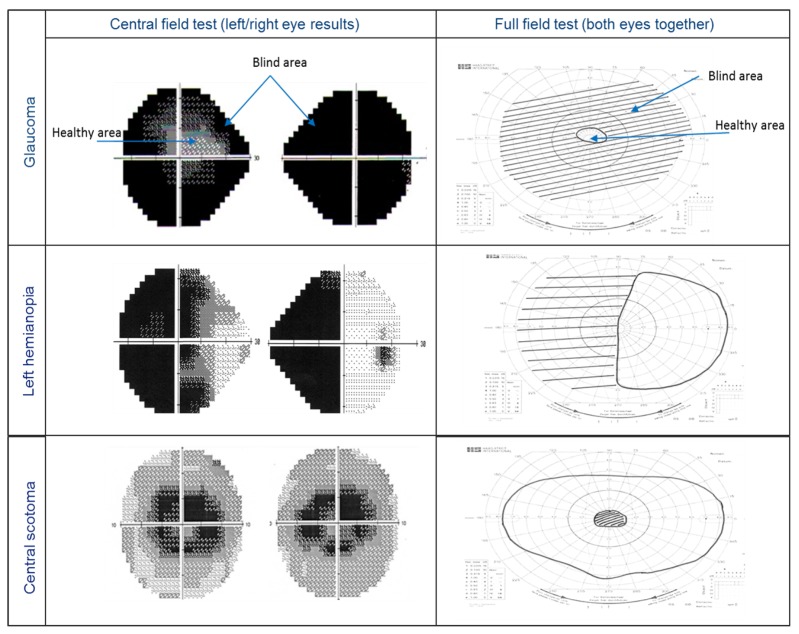
Visual field test result examples. The first row shows severe glaucoma (tunnel vision), the second row shows left hemianopia (blind to the left side, healthy in the right side) and the third row is an example of central scotoma (blind area).

Visual field test results are used to delineate both healthy and defected vision areas. Figure 5 shows three examples of visual field test results for peripheral vision defects. The left column shows the central field test (left/right eye results), while the right column shows the full field test (both eyes together). For the central visual field, the test checks the visual sensitivity for each eye’s visual field (30° around the fixation point) and displays different grey levels for each location representing different visual sensitivity. A full field test result covers ≈160°.

**Figure 6:**
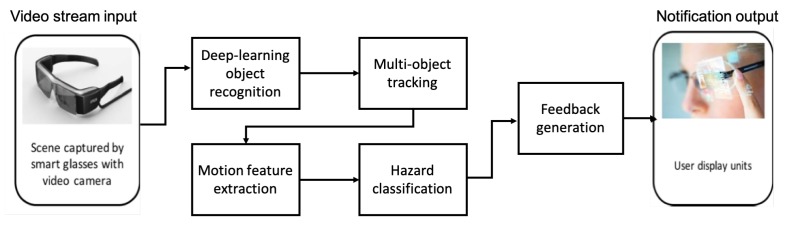
System overview including the project’s main components.

This system uses the visual field test results to search for possible threats in the user’s blind area and classifies these threats based on their danger level. The smart glasses will be used to display essential notifications outputs in the user’s healthy vision area. Figure 6 shows an overview of the proposed system and the main components used in our project.

The first stage is object detection and recognition, where objects are detected using a deep learning object classifier to determine the type and location. Motion features are extracted using the moving objects’ tracking module to determine the age (the appearance time in terms of frames), speed and direction for each detected object. This information is processed and used to determine the level of danger for each identified object using a neural network classifier.

Objects in the peripheral vision of VIP manifest themselves in different ways such as hazards, obstructions, surprises or immediate dangers. For example, someone walking in the street may not be aware of a cyclist/pedestrian walking on the other side of the road or of dangers such as a car crossing their walking route, street bollard, overhanging cables, trees or bushes to the side of the road. Not all activities in the periphery are equally important to the VIP. Therefore, a system that prioritises all these activities is needed to nudge the users to turn their head to the most immediate threat to see it through their healthy vision.

Based on the mentioned user’s preferences and needs, five hazard classes are defined using smart glasses-captured videos and public datasets. The class number represents the danger level (one is the lowest, five is the highest):

- Class 1: static object not in the user’s pathway,
- Class 2: moving objects not related to the user (any type),
- Class 3: static object in the user’s pathway,
- Class 4: person moving towards the user (or user’s pathway),
- Class 5: object moving towards the user (or user’s pathway).

The visual field has different levels of visual sensitivity depending on where the image lies relative to the fovea or fixation point [3]. This inspired us to define the user’s navigation route as the depth extent of the central vision and a small part of the macular vision (≈10°) around the fixation point. While the fixation point will vary, images are treated as centred around the fixation point.

### 3.2. Deep Learning-Based Object Detection

The first stage of the proposed system is to detect predefined objects that exist in the real world, but they are not visible to the visually impaired people. The goal of this stage is to obtain (1) the type of the detected objects and (2) current locations of these objects. In the related literature, researchers used You Only Look Once (YOLO) [33], Faster- Recurrent Convolutional Neural Networks (RCNNs) [41], Single-Shot Detectors (SSDs) [42] and other object recognition systems for object detection using deep convolutional neural networks. In our system, we found that YOLO needs a powerful graphics processing unit to perform the classification process which is not available in the smart glasses. On the other hand, the Faster R-CNNs are quite slow (on the order of seven frames per second), and this will affect the whole process of hazard classification.

A research group originally developed SSD in Google. The method can detect multiple objects at the same time in an image using a single deep neural network [42]. Since our system will be running on resource-constrained devices, we used an existing lightweight network architecture called MobileNets [43]. We used a combined version of SSDs and MobileNets, which is called MobileNets SSD. This module was trained on Common Objects in Context (COCO) dataset [44] and then readjusted on a Pascal Visual Object Classes (VOC) [45] dataset to achieve better accuracy rates.

This framework was implemented using the OpenCV 3.3 Deep Neural Network (DNN) module to create the real-time object detector that is capable of detecting 21 classes including airplanes, bicycles, birds, boats, bottles, buses, cars, cats, chairs, cows, dining tables, dogs, horses, motorbikes, people, potted plants, sheep, sofas, trains, and TV monitors. This framework was found to be the best choice to cover the object types mentioned in the user’s requirements section. In this work, a pre-trained version of the detector is used while it is planned, for our near future work, in order to re-train the classifier to reduce the number of classes based on the users’ requirements.

The detection stage starts by processing each frame to extract the objects’ blobs. These blobs are then sent to the OpenCV deep learning module to recognise the type for each detected blob. The final check is to filter out the objects with low confidence to reduce the number of false detections.

### 3.3. Multiple Object Tracking

Since the system had detected moving objects in the previous stage, the approximate location for each object is known. For each detected object, we used the location information to initialise a Kalman Filter (KF) to predict its motion over time. KF is a recursive estimator that predicts the state of the system $X_{t}$ at time *t* based on information from the previous state Xt−1 using the following equation:

(1) $$X_{t}=F_{t}X_{t-1}+B_{t}u_{t}+w_{t},$$

where $F_{t}$ refers to the state transition model that describes the change that happens to the state between time t−1 and *t*. $u_{t}$ is the vector of control inputs and $B_{t}$ is the control matrix. $w_{t}$ is the noise vector for the process transition model.

Then, the measurements’ vector $z_{t}$ is computed using the following equation:

(2) $$z_{t}=H_{t}X_{t}+v_{t},$$

where $H_{t}$ is the transformation matrix between the state vector parameters and the measurement domain and $v_{t}$ is the measurements’ noise vector. The process noise at time *t* is assumed to be Gaussian distributed noise with covariance $Q_{t}$.

The KF estimation process has two phases; the prediction and the update. In the prediction phase, the filter uses the initial estimate state $X_{0}$ and its associated variance of uncertainty (covariance) matrix $Q_{0}$ to create an estimate of the current state. For a better and more accurate estimation, the update phase computes the KF gain and uses the measurements vector from the current state to enhance the prediction result in the next state $X_{t}$.

The KF is used in this work to estimate the detected object’s location and speed. Thus, the state of each object is represented as:

(3) $$X={\left[\begin{array}{cccc} px & py & vx & vy \end{array}\right]}^{T},$$

where px, py are the centre of mass coordinates for each object and vx, vy are the velocity components.

In the prediction phase, the system predicts both the state vector $X_{t}$ and the covariance state $P_{t}$ using the following equations:

(4) $${\hat{X}}_{t|t-1}=F{\hat{X}}_{t-1|t-1}+w_{t},$$

(5) $${\hat{P}}_{t|t-1}=F{\hat{P}}_{t-1|t-1}F^{T}+Q_{t}.$$

This estimation is corrected in the next iteration (frame) after calculating the KF gain $\left(K_{t}\right)$ using the following equation:

$$K_{t}=P_{t|t-1}H_{t}^{T}{\left(H_{t}P_{t|t-1}H^{T}+R_{t}\right)}^{-1},$$

where $R_{t}$ is the observation noise covariance matrix.

Finally, the system corrects the state vector *X* and the covariance matrix *P* using the following KF update equations:

(6) $${\hat{X}}_{t|t}={\hat{X}}_{t|t-1}+K_{t}\left(b_{t}-H_{t}{\hat{X}}_{t|t-1}\right),$$

(7) $$P_{t|t}=P_{t|t-1}-K_{t}H_{t}P_{t|t-1}.$$

These two phases are applied for all detected objects over time to update the motion model for each hazard object.

As Kalman filtering is all about matrices and vectors’ operations, from the simple addition of two vectors to the inversion of a matrix, we believe that it would run in real-time applications. However, the performance of KF is highly correlated with the used processing unit. In the proposed work, we are presenting the technology that would work on a wearable device to track the moving objects. Since the object tracker is used to determine the motion model for each detected object, it is possible to skip some frames for detecting and tracking the object if we found that the process would slow down the hazard detection phase.

One of the well-known problems for tracking multiple objects at the same time is to decide which detection refers to which object. To track multiple objects at the same time, the system uses the Hungarian algorithm for best assignments between detected and estimated measurements [46]. Initially, the system defines a tracker instance for each detected object. The tracker object includes a KF and other motion features history for each identified object. The Hungarian algorithm is one of the best optimisation algorithms used to solve the assignment problem in polynomial time [46]. The algorithm also keeps track of all missing and new detections to maintain tracking consistency and efficiency.

In object tracking problems, the goal of the Hungarian algorithm is to find the best assignment that has the lowest cost between detections and tracks. The cost, in this case, represents the Euclidean distance between these two sets of variables. Each time, the system detects new objects, and the multi-object tracking algorithm updates its state to include the new/old objects using Algorithm 1.

**Algorithm 1:** Multi-object tracking update procedure.

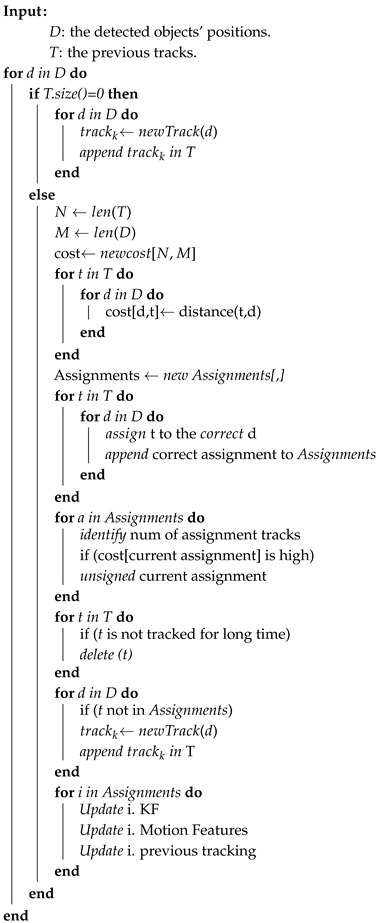



At this stage, all the detected objects have been tracked and our system can now determine the type, position and speed for each one of them. Objects with low type confidence were filtered out to reduce false alarms and increase the system’s reliability. In the next step, the motion features for each tracked object are extracted to create a hazard profile and prepare these features for the classification stage.

### 3.4. Motion Feature Extraction

The purpose of this step is to collect information about how each object is behaving while it is in the user’s environment. From the detection stage, the system recognises the type of the detected object and the confidence of that recognition and saves this information into a global feature array.

The tracker will access the same information to add the following object’s features:

1. Age: a feature that represents the appearance duration (number of frames) for the tracked object.
2. Current and estimated next location. This information is important to distinguish between moving objects and static obstacles.
3. Speed (pixels/second).
4. Motion direction.

**Figure 7:**
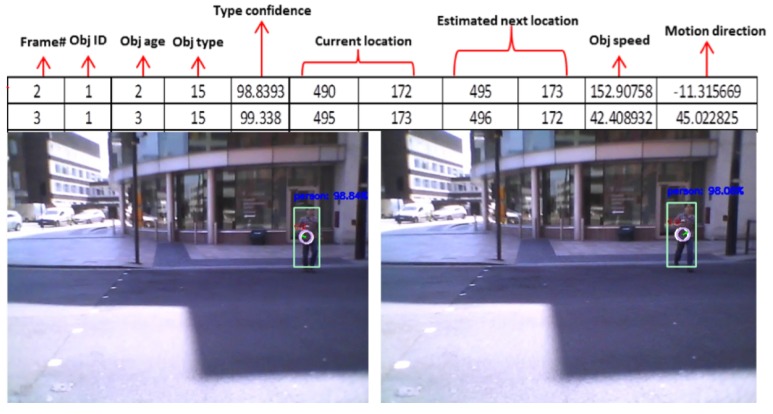
An example of the extracted features from one of our testing videos.

Figure 7 shows a sample motion features for one of our testing videos where these features were extracted over two consecutive frames. The moving object (type 15: person) is moving towards the camera. As seen, the detector recognised the object type as a person, and the tracker estimated the speed and direction for that person.

### 3.5. Hazard Classification Using Machine Learning

**Figure 8:**
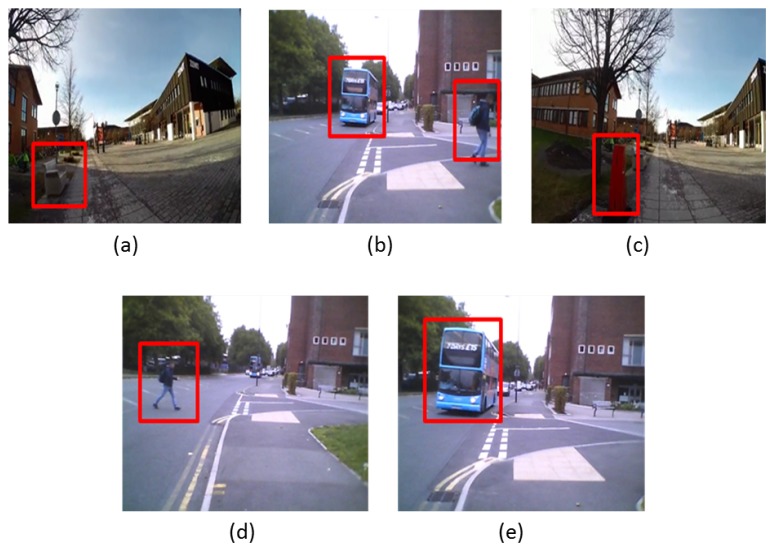
A visual example of the hazard classes. (**a**) the seat represents class 1; (**b**) the approaching bus and the pedestrian are examples of class; (**c**) the street polar is an example of class 3; (**d**) the crossing pedestrian is an example of class 4; (**e**) the approaching bus is an example of class 5.

The purpose of this stage is to classify the detected hazards to decide which object has a higher priority to notify the user. As mentioned in the users’ requirements section, the VIP needs and challenges differ in terms of the object type, motion type and other physical features. Generating feedback for each detected hazard wouldn’t be useful and may be considered to be annoying. For these reasons, we grouped the VIP choices into five hazard classes that were described in Section 3. Figure 8 shows a visual example for these classes.

These classes are based on a questionnaire in which we asked a group of visually impaired participants about the hazardous situations they face while navigating. In addition, we asked them to classify predefined hazard classes to estimate the most dangerous conditions. Based on the questionnaire results and group consultation, we defined these hazard classes.

## 4. Datasets Preparation

### 4.1. Public Datasets

In this system, it is assumed that the user will wear the smart glasses while walking indoors and outdoors during the day. Therefore, it was important to test the performance of the system using a moving camera with variable weather and light conditions.

In the evaluation part, we used two types of datasets to test the accuracy of the hazard classification and the object detection and tracking stages. We first tested our system using the publicly available dataset from the Cambridge-driving Labeled Video Database (CamVid) [47,48]. We chose this dataset for two reasons: (1) it was captured using a fixed camera on a moving vehicle to show the drivers view and (2) it contains a set of different objects that our object recognition system is trained to detect. Unfortunately, we could not have the field of view (FOV) information for the used camera in this video. Thus, we assumed a standard dash camera FOV of 120°.

In the experiments, the system is tested on the sequence Seq06R0 that contains high-quality 30 Hz footage captured in street view. It shows a video taken while the vehicle is moving amongst other vehicles (moving and stationary). Some of these vehicles are moving towards the camera, and others are moving away. In addition, some pedestrians are seen crossing the road and walking aside the moving vehicle.

### 4.2. Private Datasets

For more realistic evaluation covering all possible hazard classes, we decided to create our datasets for training and evaluating the system to check the performance using a wearable camera with different conditions (light changes, object deformation, and object occlusion). We used Moverio BT-200 smart glasses (Long Beach, CA, US) to capture in-street videos with the help of a normal vision participant (Indoor and outdoor video capture is in accordance with the ethical approval from the Research Ethics Committee at the Faculty of Science and Engineering, University of Liverpool, UK. Reference: 1982).

**Figure 9:**
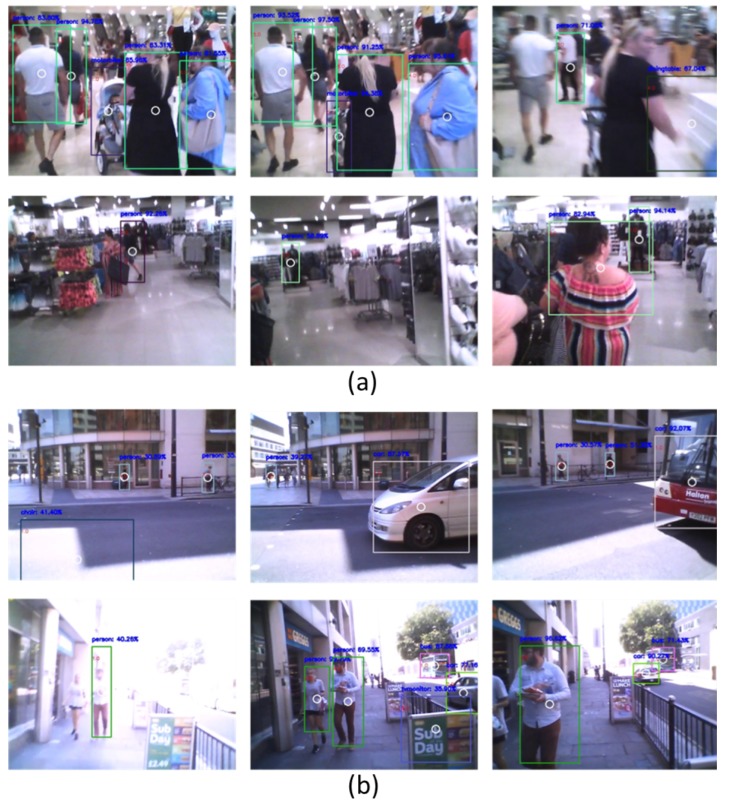
Examples captured by the Moverio BT-200 smart glasses. (**a**) indoor video frames; (**b**) outdoor frames.

For this purpose, we captured two indoor videos with a total of 23 s (644 frames) and seven outdoor videos with a total of 221 s (6188 frames) using the Moverio BT-200 (≈28 Frame Per Second (FPS)). Figure 9 shows some examples of indoor and outdoor videos. In these examples, the blurriness and the deformation of the detected objects can be seen. This is due to the camera movement and shakiness while the user is moving. For each detected object, its hazard class was marked by an expert to be used in the training and testing stages.

### 4.3. Feedback Generation

**Figure 10:**
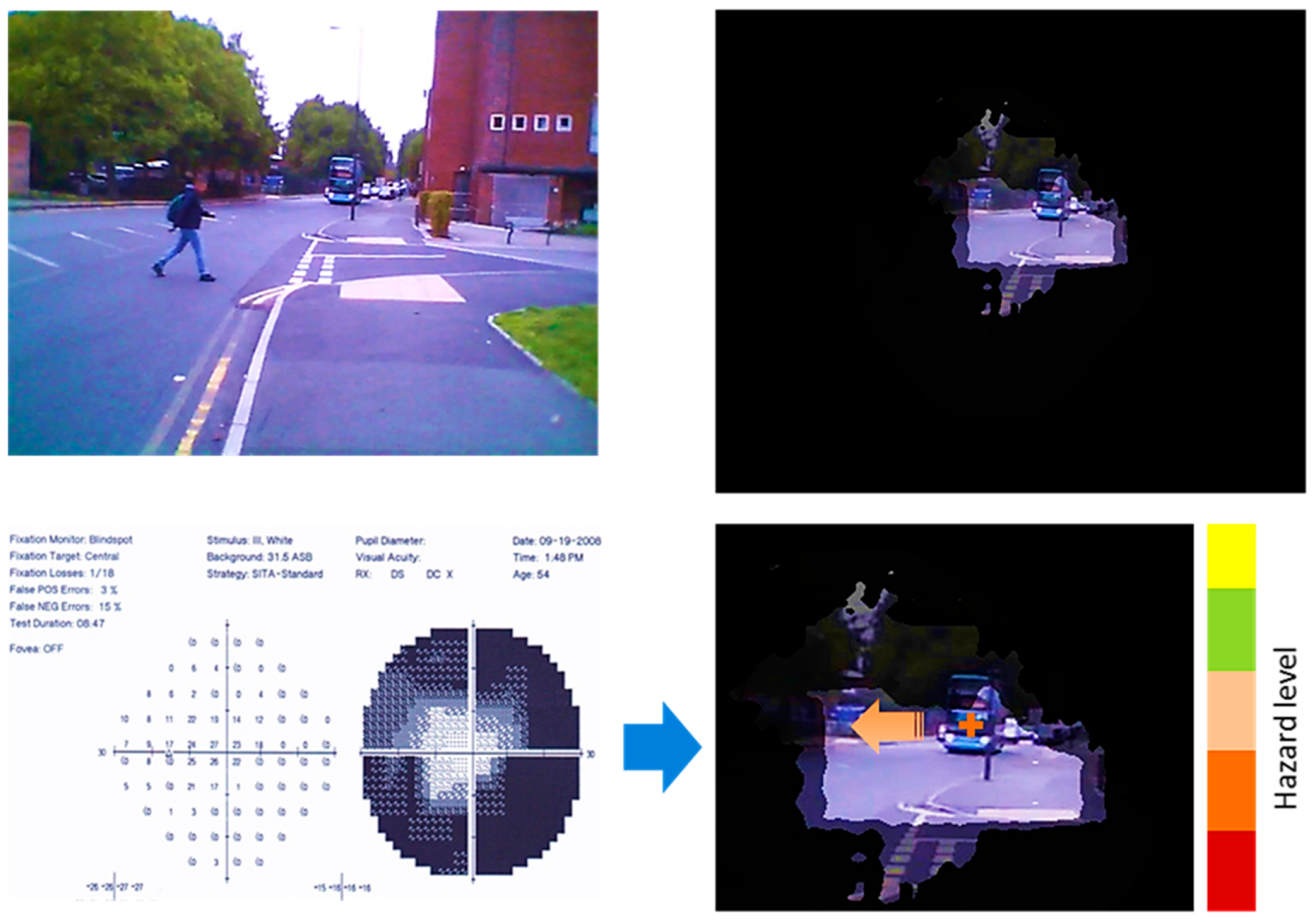
Feedback generation based on the visual field test. (**top left**) input video frame; (**bottom left**) visual field test result; (**top right**) the frame as viewed by a tunnel vision patient; (**bottom right**) sample visual notifications shown on the healthy vision area.

The final step before producing the hazard feedback is to generate a suitable notification output. As described previously, the classified hazards are provided in the form of visual notifications within the user’s healthy vision as shown in Figure 10. The top left image shows the original video frame from the private dataset. The top right image shows how a tunnel vision patient would see the same scene. The bottom left image is the visual field test result as described in Section 3. The bottom right image shows an example of output from our system on the top of the user’s healthy vision area.

The system detected and tracked two object; the Moving bus and the moving person to the left side. Based on the motion model for these objects, the system classified them into class 5 (red notification-high danger level) for the bus and class 4 (orange notification-high danger level) for the person. Since the bus is visible for the user in the healthy vision area, the system generates only one notification for the moving person to the left side. The scene has been magnified for demonstration purposes. The notification generation and the visual interpretation stages will be investigated further in our near future research work through an explanatory study with VIP group.

## 5. System Evaluation and Output

This work is part of a larger project for developing a user-centred, wearable assistive device for people with visual field defects. In this paper, we presented an assistive technology for people with peripheral vision loss. Therefore, we analysed the performance for the hazard detection and classification subsystems and evaluated the feedback generation module based on users’ recommendations. We implemented the proposed system on the Moverio BT-200 smart glasses which captures 15 FPS. videos and the average processing time of a single frame at the glasses is 0.49 s. Based on this, the glasses can process at least 2 FPS, which means that we have to reduce the input frame rate to guarantee real-time feedback generation. Thus, the glasses are processing one frame per seven captured frames without affecting the overall detection accuracy, and is considered sufficient for the purposes of this paper.

The presented evaluation in the paper was performed on a MacBook laptop (2.7 GHz Intel core i5 processor, 8 GB RAM) (Cupertino, CA, USA) which was able to process the high resolution CamVid videos (30 FPS) with an average of 0.2160 s per frame and an average of 0.1932 s for videos captured by the Moverio BT-200 smart glasses.

A three-layer NN model was created with seven inputs to the input layer representing the detection and motion features: object type, detection-type confidence, object age, object location, object speed, and motion direction. The output layer has five nodes representing the five hazard classes. For each detected object, the classifier decides its hazard class based on its motion features. Some of these objects may change its class over time depending on the way it is moving around the user. For each object, its class is determined for every frame in which the system detects it.

The average MSE for each of the ten experiments is calculated to evaluate the performance per specific number of hidden neurons using the following equation:

(8) $$MSE=\frac{1}{N}\sum_{i=1}^{N}{\left(p_{i}-o_{i}\right)}^{2},$$

where *p* is the predicted value, *o* is the observed value and *N* is the total number of values.

The datasets described in Section 4.2 are used to evaluate the classification model. A total of 3536 samples are used, and the best NN configurations were found to provide the lowest False Positive Rate (FPR) and the highest True Positive Rate (TPR) for all the hazard classes using 19 hidden layers and 0.3 decision threshold. The best NN configuration is found to provide the highest TPR of 90% with the lowest FPR of 7%. An average of 13% False Negative Rate (FNR) is achieved.

**Figure 11:**
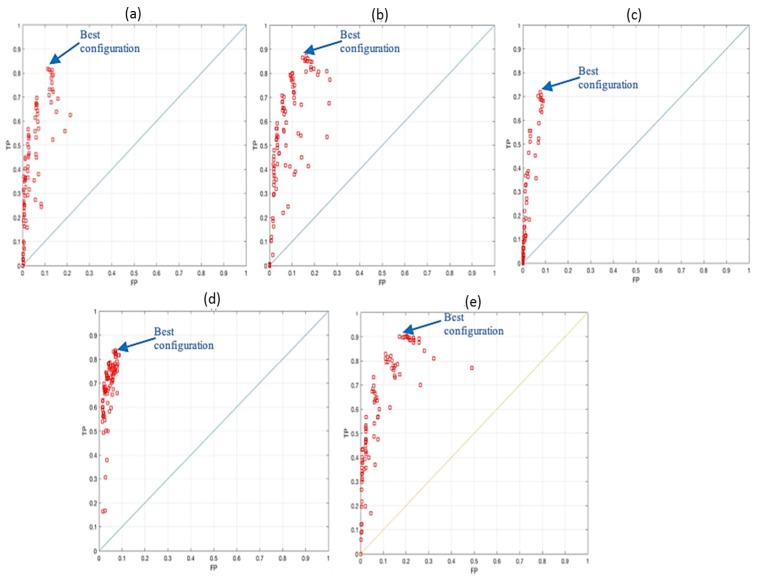
ROC curves for the NN optimisation. (**a**) results for class 1; (**b**) results for class 2; (**c**) results for class 3; (**d**) results for class 4; (**e**) results for class 5.

In this system, the TPR represents the rate of truly detected, tracked and classified hazards compared to the total number of classifications. The FPR represents the rate of falsely providing a hazard notification (false alarm). The FNR represents the rate of cases that the system reported it is not dangerous while, in fact, it is. The average MSE value for the five classes is 8.7655%. Figure 11 shows the Receiver Operating Characteristics (ROC) curve for the five classes. These results are very good as this is the first version of our system. However, it could be improved in the future using more useful features for modelling the hazard classification process.

The regression analysis has been applied to the classification results to understand the relationship between the predicted hazard class (dependent variable) and the extracted features (independent variables). The best testing coefficient of determination was R = 0.72, meaning that the regression model can reasonably predict the hazard class perfectly.

These results are promising and could be used to determine hazard classes in real-time applications to help people with impaired vision in their daily activities.

**Figure 12:**
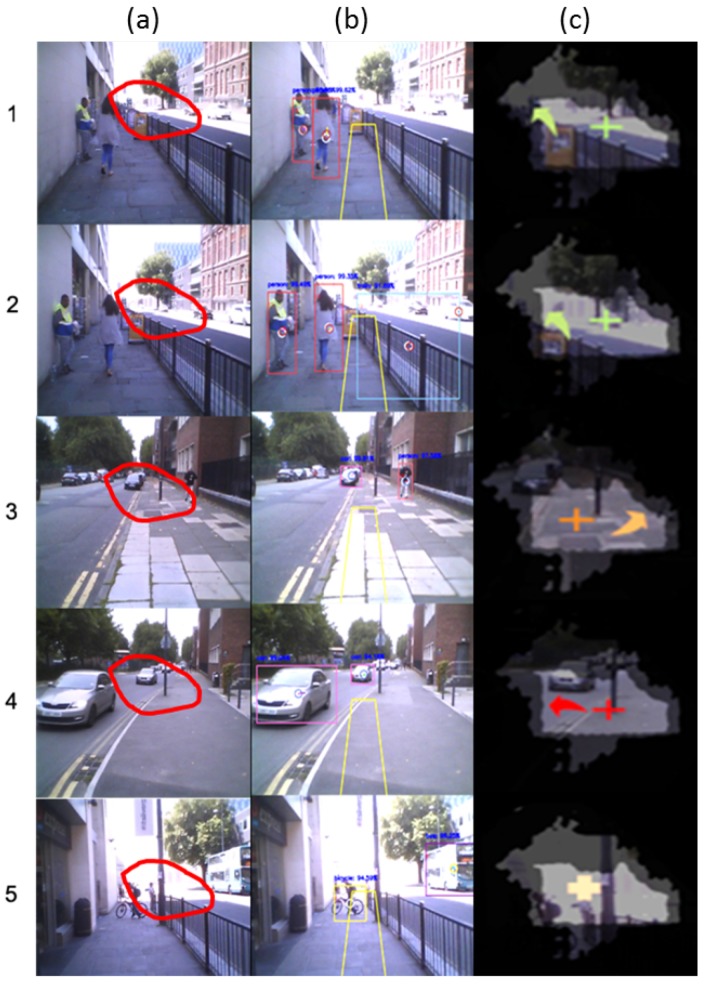
Examples from the feedback generation stage for the defined hazard classes.

Figure 12 shows some examples of the feedback generation stage. Column (a) represents the captured frame. The redline shape overlapping with the image defines the seeing area for a patient with severe glaucoma condition mentioned in Figure 5. Column (b) shows the hazard detection and tracking modules results. Yellow lines show an approximate illustration for the user’s navigation route. These results are fed to the NN classifier to determine the hazard class. The result of the classification module is displayed in column (c).

In the first row, two objects are detected and tracked; stationary man (class 1) and a moving woman (class 2). Since class 2 is more dangerous than class 1, and the user is unable to see both of them, the system generates a green arrow pointing to the left side (according to the reference cross symbol in the centre of the healthy area). In the second row, two new objects are detected to the right side of the user; stationary train (class 3) and a moving car (class 2). Although class 3 has a higher priority, the system ignores this because the user can see this object (2b) and thus generates a notification for the class 2 hazard. In this case, the notification is for the moving woman, not the car since its position is closer to the user. It is worth mentioning that the wrong object type (train instead of a wall) is due to false detection.

In the third row, the system detected a stationary car in the user’s navigation route (class 3) and a person moving towards the user (class 4). Since class 4 has higher priority than class 3 and the user can see the class 3 hazard in the seeing area (3b), a notification is generated for the class 4 hazard as an orange arrow pointing to the right side. Class 5 (highest priority) is detected in the fourth-row example as two cars coming towards the user. The system generates one red arrow pointing to the left side that shows a closer car). Finally, class 3 hazard notification is shown in the fifth-row example as a bicycle is detected in the user’s navigation route. Although the user can see this object, the system produces a simple notification (cross symbol with light orange colour) without any arrows to notify the user to look straight because there are no other dangerous hazards in the scene.

Currently, the implemented system generates a single notification for the highest hazard level as a visual notification (arrows with different colours reflecting the hazard type and pointing to the object direction). A participant study was conducted with a group of patients with different visual field defects to explore their preferences, suggestions and opinions about the notification style, frequency and other presentation features.

After describing the project’s idea and design, basic demography information and the visual impairment history of the participants were collected. We found that 100% of the participants use portable devices like the iPhone and iPad and only 20% of them use these devices for navigation. Regarding the feedback format, the participants prefer to use either visual notifications (33%) or hybrid style (66%) [visual and vibration (75%), visual and beeps (25%)]. The participants tried the Moverio BT-200 smart glasses with basic notifications in an indoor environment. Due to the ethical approval constraints, we were unable yet to perform outdoor experiments for the proposed system. Therefore, we presented the basic system concepts (a single notification for the highest hazard level) to the participants and collected their feedback through a questionnaire, and the results will be analysed and used for the ongoing developments and will be published in an extended study in the near future. In general, we believe that the participants were extremely happy and satisfied with the technology and thrilled to try more features soon.

## 6. Conclusions

This paper presents a novel context-aware hazard attention system to be used on smart glasses to help people who suffer from peripheral visual field defects. The system includes hazard detection and recognition, hazard tracking and real-time hazard classification modules. Based on the detected motion features, the system assigns a hazard type class for each detected threat in order to generate a suitable visual notification output.

The main goal of this system is to increase the user’s awareness of the surrounding environment without interfering with healthy vision. Unlike other obstacle avoidance and navigation systems, the system is directed to the people who have partially healthy vision. Our system uses this healthy vision and augments it by new, meaningful and smart notifications that appear only if necessary. This system has been tested on both publicly available and new private datasets. The classification stage shows promising results and the system can truly classify any detected hazard into one of five predefined hazard classes.

Through our research collaboration with the Department of Health Services Research, we created our own dataset for hazard detection and classification. Using Epson’s Moverio BT-200 smart glasses, we captured indoor and outdoor videos and an expert labelled them to one of five hazard classes. These classes were discussed with a visual field loss patient group.

We had a group meeting with the mentioned patients’ group to discuss the idea of the proposed technology and to gather their requirements regarding the feedback generation stage according to their personal experience. The participants tried the glasses and discussed the project’s idea and stages and gave us their feedback. Some of them accepted to perform real-world experiments in future. In general, we believe that the participants were extremely happy and satisfied with the technology and thrilled to try more features soon.

Our next research directions will focus on adding more features for better hazard model representation and personalising the notification style and position using the user’s visual field test results. In addition, we will use some of the state-of-the-art smart glasses with the newest technologies in the field.

## Abbreviations

The following abbreviations are used in this manuscript:

AT	Assistive Technology
AR	Augmented Reality
CamVid	Cambridge-driving Labeled Video Database
FOV	Field Of View
FPR	False Positive Rate
FPE	Frame Per Second
KF	Kalman Filter
MSE	Mean Square Error
NN	Neural Network
SSD	Single Shot Detector
TPR	True Positive Rate
VIP	Visually Impaired People
VR	Virtual Reality
YOLO	You Only Look Once
